# Exostosis Eburnea

**Published:** 1889-04

**Authors:** D. L. Peeples

**Affiliations:** Navasota, Texas


### EXOSTOSIS EBURNEA.

By D. L. Peeples, M. D., Navasota, Texas.

[For Daniel's Texas Medical Journal.]

About the 19th of October last, my attention was called to the consideration of a rapidly growing osseous tumor, upon the external tuberosity of the dextral femur on a lad of this place. Patient æt. 12 years, height 4 feet, 6 inches, weight 65 pounds; complexion pale and sallow, constitutionally frail and lean, but of a sanguine temperament, with no cardiac lesions, except force and volume comparatively feeble and small. After investigating the clinical history of the case, I concluded it was of syphilitic origin, (hereditary.) Dimensions of tumor, 1½ inches broad, 3¾ inches long, and protruding 2¾ inches from fermur. Ossification began above the tuberosity, and had extended down to infra-condyloidal border, sufficiently to impair articulation, and he constantly suffered nocturnal pains, and was growing much more inconvenient and uncomfortable. Early operation for removal of tumor was advised. .

On the 23rd of October, I put patient on the following:

| | | |
|---|---|---|
| ℞ | Hyd. Chlor. Corr. | gr. ii. |
| | Pot. Iodide, | ʒvss. |
| | Fer. et Pot. Tart. | ʒv. |
| | Tr. Columbo | ℥i. |
| M | Syr. Simplex q. s. | ℥viii. |

Sig.—Teaspoonful in water three times per day, for purpose of preparing him for the operation. My two assistants were Drs. Ketchum and Gay. The operation was performed nnder the latest and strictest antiseptic precautions. Solution for irrigation, hyd. chlor. corr. 1 to 3000. Solution for instruments, acd. carb. 1 to 20. Esmarche's bondage and tourniquet were used, hence hemorrhage was reduced to the minimum. Ether was used instead of chloroform. After removal of bandage, I made an incision 4½ inches long, from below upwards, dissected the parts, exposed tumor and extirpated same. When the tourniquet was removed, considerable oozing occurred, but was checked readily by hot water sponge packs, after which wound was cleansed and filled with iodoform; drainage tubes adjusted, and the parts brought together, dressed with woolwood, and patient aroused without trouble. The whole work was done in one hour and ten minutes. About 1½ hours after operation, patient collapsed gravely, but was rescued by administration of whisky and digitalis, hypodermically, whisky and amm. carb. internally.

I gave quinine and strychnine regularly three times daily. Quinine in the elixir was only about 1 or 2 grs., but in addition he took 5 grs. (quinine), every three hours, for first two days, when I gave 5 grs. every four hours; but, as he improved, it was reduced to every five hours, and finally every six hours, until discontinued. In addition to above, he took 15 M. syr, iodide fer. Close attention was paid to bowels. Pulse ranged from 75 to 100, temperature from 99 to 101 for first few days, when they became normal. Wound was redressed on next day, on account of little hemorrhage the first night, and redressed as often as necessary. Listerine was passed from one tube through the wound out the other. No opiates or anodynes were used during the entire course of treatment, the absence of pain being a conspicuous feature. Suppuration just enongh to slightly stain dressing at extremity of tubes; nausea, vomiting, cephalalgia, and cardial weakness were the principle points to guard against. On seventh day, tubes were removed, and on fourteenth day patient was up and walking about; though the whole knee became œdematous, and I requested him to take his bed, which he did, until the 25th of December, on which day I accompanied him to Montgomery.

There was slight stiffness of the joint only for a short while. Patient has not had any nocturnal pain whatever, since the operation. The operation was performed December 2, 1888. I have one piece of the bone, which weighs one ounce, the balance was taken away by small pieces.

It is scarcely necessary to add that the very important feature of nutrition was carefully attended to, patient being supported for the first few days by nutritive enemata, and so soon as the powers of assimilation and digestion were recuperated, patient was fed liberally in small quantities only, at first, on milk, beef essence and meat juice, and other such nutritives. The usual means of suppressing or obviating nausea, were resorted to, of course.

Patient is now at school, and walks four miles daily, two miles each way; besides, he plays and romps without any inconvenieuce.

